# Supplementary material for: Patterns of Intron Gain and Loss in Fungi
Source: PLoS Biol. 2004 Nov 30;2(12):e422. doi: 10.1371/journal.pbio.0020422 (PMC532390; doi:10.1371/journal.pbio.0020422)
Supplement: Table S1 — Also available at http://genes.mit.edu/NielsenEtAl/. (4.3 MB ZIP). [file pbio.0020422.st001.zip › NielsenEtAl/html/5.html]

AN5564.1.NCU04924.1.MG00099.1.FG07907.1


```
 CLUSTAL W (1.82) Multiple Sequence Alignments - Introns Inserted


Sequence 1: MG00099.1	535 aa
Sequence 2: FG07907.1	474 aa
Sequence 3: NCU04924.1	526 aa
Sequence 4: AN5564.1	483 aa
Alignment Length: 538 aa
Number Identitical Residues: 247 aa
Alignment Score (without introns) 11792


MG00099.1 	-MPAFADIDFGVDPNPTGPPSKTNGTTNGHTNGHSNGTNGHTNGNGVAHATNSTAALEDA
NCU04924.1	MSGQQSDIVVGIPNPNTKTNPHTP--PESPTETQIKALDGVEKQQQQEQPQQLNVAKTRG
FG07907.1 	---------------------------------MSN-ANGESNG----------------
AN5564.1  	-MPRFEDSDFGVEGSPSAPPARGS-----TLDTRSPGDRGSMGG----------------
          	      .   .     : . .          .    .  *                    

MG00099.1 	LSPRSILPIPGPDGNGEEPSDPIEVPATRPSVSDAEKYMHNLSMSPSMRDRRGSRNSFGT
NCU04924.1	NSRQDSLTIPLSP--SVANHNTIEVPATRSPDAHADLYMHNLSASPSLKERRLSRNSFGA
FG07907.1 	VDHRESLAIPG----TEEGNGPQEIPATRSSISEAAKYMHTLSVSPSMRDRRSSRNSFGT
AN5564.1  	-----QLPIPN------DANNIVEVPATRSSISDAASFMHNLSLAPSSRDRRGSRNSFGT
          	      *.**          .  *:****.. :.*  :**.** :** ::** ******:

MG00099.1 	SLPIPRIHRRSRLSSVHRPSEEEGSKSSRP-VMPVLQSSRHIIQSQIQDVQAAKVKKVRN
NCU04924.1	SLPIPRSKRQSRLSSVTFPGGVAEALQEASGGTLPLKPTREFLADRVQDIHSEKVEKVKN
FG07907.1 	ALPIPRK-RQSRLSSVHHAD----GRPTRP-GMPPIQPTRDLLIAQVQDQMADKVKKAKN
AN5564.1  	SLPIPRSPRVSRLSSVVPAD-----------------VSRDILASQIQDMSKEKVAAAKN
          	:*****  * ******  ..                  :*.::  ::**    **  .:*

MG00099.1 	MAFAFDIDGVLVHGDRLIPEGKRVLEILNGDNELGMKIPHIFLTNGSGKPEQARCDQLTK
NCU04924.1	MSFVFDIDGVLVHGDRLIPEGKKVLDMLNGDNELGIKIPHIFLTNGSGKPELARTEQLSK
FG07907.1 	MAFAFDIDGVLVHGDRLIPEGKKALEILNGDNELGIKIPHIFLTNGSGKIEKARCEQLEK
AN5564.1  	MAFAFDIDGVLAHGNHPIEEAKQALKMLNGDNELGIKIPYILLTNGGGKTEAARCQQLSE
          	*:*.*******.**:: * *.*:.*.:********:***:*:****.** * ** :** :

MG00099.1 	ILGSKVDTAQFIQSHTPMSALAAYYGTVLVVGGEGTKCRDVAELYGFKDVVVPNDIVAWD
NCU04924.1	ILQNPISTDQFIQSHTPMRALAEYYNTVLVVGGEGYKCREVAEEYGFKDIVVPNDIVAWD
FG07907.1 	ILGNPVSTDQFIQSHTPMSALSEYYSTVLVVGGEGYRCREVAEQYGFRNIIVPNDIVAWD
AN5564.1  	VLDCPISTDQFIQSHTPMQALSDYYETVLVCGGEGTKIREVAENYGFKNVILPKDIQAWD
          	:*   :.* ********* **: ** **** **** : *:*** ***:::::*:** ***

MG00099.1 	PTIAPYRVFTDEERKHARPRDFSQTNIEAILVFSDSRDYATDMQIIMDLLRSKDGRLHTM
NCU04924.1	PSIAPYRVFTDEERATSRPRDFTKTNIEAIMVFSDSRDYATDMQIIVDLLRSENGRLGTV
FG07907.1 	PTIAPYRVFTDEERATSRPRDFTKICIEAIMVFSDSRDYATDMQIIMDVLRSKNGRLGTV
AN5564.1  	PTVSPWGHFSEQDRAEAKPRDFSKIKFDAILVFADSRDYQTDFQIIMDLLLSEDGRLLTR
          	*:::*:  *::::*  ::****::  ::**:**:***** **:***:*:* *::*** * 

MG00099.1 	CRDDPVSNRIPIYFSQGDMLCPTEHPIP-RMSQGAFRIGLEAMYKSL0TGVDLERTVYGK
NCU04924.1	A-KDPVSQRIPIYFSQGDLLCPTEHPTP-RMSQGAFRIGLEAMYKAL~TGVDLERVVYGK
FG07907.1 	A-EDPVAERVPIYFSQGDLLCPTEHPTP-RMSQGAFRIGLEAMYKAL~TGADLERVVYGK
AN5564.1  	A-KDLTQGRIPIYFSQGDLIMPTDHKGPTRLTQGLYRISIEAQYKAL~TGLDLERVVYGK
          	. .* .  *:********:: **:*  *:*::** :**.:** **:* ** ****.****

MG00099.1 	PELATYKYADEVLTSWMGNLYGEEEERLPENIYMIGDNPASDIVGGNMYGWNTCLVRTGV
NCU04924.1	PEMATYKYADEVIASWMEQIHGEE--KIPQNIYMVGDNPASDIVGGNLYGWNTCLVRTGV
FG07907.1 	PELATYKYADDVLTSWMGELHGEE--RLPENIYMIGDNPASDIVGGNMYGWNTCLVRTGV
AN5564.1  	PEEATYKYADEVLRSWMEQIHNEN--TLPENIYMVGDNPASDICGGNMHGWNTCLVRTGV
          	** *******:*: *** :::.*:   :*:****:******** ***::***********

MG00099.1 	FQGGDNDEENPANFGVFKNVLEAVSTALRKELGDDFKLNFDEHSINPIKACPDSAAAIE
NCU04924.1	FQGGENDEENPANFGVFANVWEAVTSACRKELGDDFKFKWDDRVN-PLN---INASAIE
FG07907.1 	FQGGDNDENNPANFGVFPNVLEAVKKALRAQMGDDFKCYFDEKINPVLHGDANDAAAVV
AN5564.1  	FQGGDNDETNPANFGVFPNVLEAVKAAVRKELGQEFKLKWNPKVNPVLHG--NNASAIE
          	****:*** ******** ** ***. * * ::*::**  :: :    ::.   .*:*:
```
